# Treatment of Uterine Myoma with 2.5 or 5 mg Mifepristone Daily during 3 Months with 9 Months Posttreatment Followup: Randomized Clinical Trial

**DOI:** 10.1155/2013/649030

**Published:** 2013-07-29

**Authors:** Josep Lluis Carbonell, Rita Acosta, Yasmirian Pérez, Roberto Garcés, Carlos Sánchez, Giuseppe Tomasi

**Affiliations:** ^1^Mediterránea Médica Clinic, C/Salvador Guinot 14, 46017 Valencia, Spain; ^2^“Eusebio Hernández” Gynecology and Obstetrics Teaching Hospital, Avenue 31, Calle 82, Marianao, 11300 Havana, Cuba; ^3^University of the Basque Country, 48014 Bilbao, Spain

## Abstract

*Objectives*. To evaluate the efficacy, safety, and quality of life by using 2.5 and mifepristone 5 mg daily doses to treat uterine fibroids over 3 months with a 9-month followup period. *Design*. Randomized clinical trial. *Place*. “Eusebio Hernández” Hospital, Havana, Cuba. *Subjects*. 220 women with symptomatic uterine fibroids. *Treatment*. One-half (2.5 mg) or one-whole 5 mg mifepristone tablet. *Variables to Evaluate Efficacy*. Changes in fibroid and uterine volumes, in symptomatic prevalence and intensity, and in quality of life. *Results*. After 3-month treatment, fibroid volume decreased by 27.9% (CI 95% 20–35) and 45.5% (CI 95% 37–62), in the 2.5 and 5 mg groups, respectively, *P* = 0.003. There was no difference in the prevalence of symptoms at the end of treatment, unlike after 6- and 9-month followup when there was a difference. Amenorrhea was significantly higher in the 5 mg group, *P* = 0.001. There were no significant differences in mifepristone side effects between the groups. Both groups displayed a similar improvement in quality of life. *Conclusions*. The 2.5 mg dosage resulted in a lesser reduction in fibroid size but a similar improvement in quality of life when compared to the 5 mg dose. This trial is registered with ClinicalTrials.gov NCT01786226.

## 1. **I**ntroduction

The selective progesterone receptor modulators (SPRMs) are proving to be a new source of hope in treating uterine fibroids [1, 2]. Recently, ulipristal acetate has been authorized for use in the European Union in 5 mg daily doses over 3 months to improve symptoms in presurgery patients [3, 4]. The oldest, almost pure antiprogestin, mifepristone, has shown great effectiveness with different dosages in multiple studies into the treatment of this condition [5, 6]. Mifepristone in 5 mg doses has proven itself to be an efficient and safe therapeutic medicine as well as achieving an observable improvement in quality of life [5–10]. Eisinger et al. in a 17-case pilot study using 2.5 mg doses of mifepristone obtain lesser reductions in uterine volume, but a similar quality of life in comparison with 5 mg mifepristone [11]. Feng et al. demonstrate that the improvement in quality of life obtained with 2.5 or 5 mg doses of mifepristone is partially related to the reduction in symptoms, particularly pain and bleeding, but bears no relationship with reduction in uterine volume [9].

The aim of this study is to evaluate the effectiveness, safety, and quality of life obtained using 2.5 versus 5 mg mifepristone daily over 3-month treatment and 9-month followup. 

## 2. Material and Methods

### 2.1. Design

This randomized clinical trial consisting of two treatment groups was approved by the Scientific Committee of the “Eusebio Hernández” Gynecology and Obstetrics Teaching Hospital. Subjects were recruited from the gynecological hospital classification consultancy and primary health care units. All subjects gave their informed consent to participate in the study. The clinical trial was carried out in accordance with the revised version of the Helsinki Declaration and with the standards of Good Clinical Practice. The study began in March 2010 and the last subject to be included was evaluated in March 2012, nine months after termination of treatment with mifepristone.

### 2.2. Subjects

Female volunteers, 18-year old or older, with uterine fibroids were eligible for the study. Inclusion and exclusion criteria were the same as those used in a previous study of ours [8]. 

### 2.3. Treatment

Group  I: one half (2.5 mg) of a 5 mg tablet of mifepristone was taken orally every day for 3 months.
Group  II: one whole 5 mg tablet of mifepristone was taken orally every day for 3 months. The mifepristone was supplied by Litaphar Laboratory, Guipúzcoa, Spain.

### 2.4. Examinations Performed

Complete gynecological examination and abdominal or vaginal ultrasound examination of the uterus at the beginning and end of treatment and at 3, 6, and 9 months after termination was performed. Fibroid volume was calculated using the formula: 0.524 × *A* × *B* × *C* where *A*, *B*, and *C* are the diameters of the sphere in each of the 3 planes and are expressed in cubic centimeters [12]. If the subject had more than one myoma, the measurement of the biggest was taken and its variations were used to evaluate efficacy. The volume of the uterus was measured using the previously mentioned formula. Ultrasonography was used to calculate endometrial thickness in mm. All ultrasound calculations were carried out with ALOKA Co. Ltd. Ultrasound Diagnostic Equipment SSD-4000, Mitaka-shi, Tokyo, Japan and two doctors specialized in ultrasound carried out the measurements. Calibrations taken at different stages of the study were performed with the sonographers ignorant of previous measurements, knowing only the localization of the myoma to be measured.

Blood samples were taken for hematological tests and hepatic function at the initial consultancy session and 3 months into treatment; furthermore, hemoglobin was evaluated during the followup after 3, 6, and 9 months. It was decided beforehand that any subject presenting alterations in transaminases 3 times or more above their normal maximum limit, in line with FDA recommendations, would be excluded from the study [13]. 

Prior to treatment samples were taken from all subjects for cervical cytology and an endometrial biopsy was performed if any of the following criteria applied: (a) endometrial thickness >8 mm, (b) episodes of vaginal bleeding lasting more than 10 days, (c) vaginal bleeding during the three weeks prior to onset of menstruation, and (d) copious vaginal bleeding; (2) at 45 days of treatment if at least one of the conditions mentioned above was present; and (3) to all women once treatment was over. Horne and Mutter et al. criteria were used to interpret the biopsies [14, 15].

### 2.5. Control Visits and Evaluation

During treatment, there were control or evaluation visits every 45 days. Once treatment was over, the subjects were evaluated 3, 6, and 9 months later. During this period no other treatment or placebo was administered that might shroud the fibroid evolution or symptoms, and thus any chance of a placebo effect as a possible explication of an improvement sustained in the prevalence and intensity of symptoms was eliminated. 

### 2.6. Variables to Evaluate Efficacy

The main variables to evaluate efficacy were the percentage changes in fibroid and uterus volumes before starting, 3 into treatment and 3, 6, and 9 months after its termination. Other variables used to estimate efficacy were changes in the prevalence of pelvic pain, lumbar pain, rectal pain, pelvic pressure, urinary symptoms, dyspareunia, hypermenorrhea, and metrorrhagia. Also, pelvic pain intensity and hypermenorrhea were evaluated by a visual analogue scale (VAS) from 0 to 10 where 0 represented absence of symptoms and 10 represented their maximum value and was indicated by the patient herself. All these variables were measured in each of the study evaluation periods.

### 2.7. Variables to Evaluate Safety

(a) Changes in endometrial thickness: an evaluation was undertaken at the beginning, every 45 days during treatment, and every three months up to a maximum of 9-month posttreatment monitoring. (b) Mifepristone side effects: amenorrhea, hot flushes, nausea, dizziness, vomiting, fatigue/tiredness. (c) Variations in aspartate-aminotransferase (ASAT) and alanine-aminotransferase (ALAT) values before treatment began and upon its termination. (d) Frequency of endometrial changes associated with selective progesterone receptor modulators (SPRMs). Amenorrhea was defined as the absence of bleeding greater than 6 days spotting. 

### 2.8. Quality of Life

This was evaluated by means of the UFS-QOL (Spies) test using a scale of 1 to 100 points to indicate improvement in quality of life. The questions in this test are grouped in 8 different sections with reference made to various aspects: sexual activity, self-control, energy and mood, and so forth, where an increase in score means an improvement in the quality of life and then another area dealing with symptoms where a reduction in score represents an improvement in the subject's quality of life [16]. 

### 2.9. Number of Subjects to Be Included

The expected reduction in fibroid volume was the variable used to calculate the size of the study sample. It was assumed that at the end of treatment with 5 mg mifepristone the average fibroid volume would be 20% less than that obtained with 2.5 mg. A power analysis indicated that with 101 subjects in each treatment group, 202 in total, it was possible to detect that difference with an error of Type I = 5% and with a minimum power of 90% in a unilateral hypothesis [17]. The study sample size was increased by 8%, (110 patients in each group, for a total of 220 in the study as a whole), so as to offset subject loss over the course of the treatment or during the relatively long posttreatment followup period. 

### 2.10. Assignation to Treatment Groups

People not participating in the study prepared sealed opaque envelopes containing a card bearing the text “mifepristone 2.5 mg” or “mifepristone 5 mg.” Once the subject had been evaluated in line with the inclusion and exclusion criteria and had signed the informed consent, the envelope corresponding to the subject's numbered incorporation into the study was opened and she was included in the treatment group indicated on the card contained in the envelope. The study was not blind; thus, both doctor and patient knew the mifepristone dosage administered. 

### 2.11. Presentation of Results and Statistics

The results are presented in absolute frequencies, percentages, averages, standard deviations, and 95% confidence intervals for the average fibroid and uterine volumes. Pearson's chi-square test, the Student's *t*-test for independent samples, and normal approximation for proportions were used to evaluate homogeneity between the two treatment groups. Comparisons between treatment groups regarding the continuous variables of effectiveness and safety were carried out using the Student's *t*-test for independent samples and the normal approximation for proportions to compare prevalence of fibroid symptoms and incidence of mifepristone side effects in each evaluative period. In all cases, *P* < 0.05 was considered significant and all tests were two tailed. Data was processed with the SPSS 11.5.

## 3. Results

### 3.1. Inclusion and Adherence to Treatment

**Figure 1:**
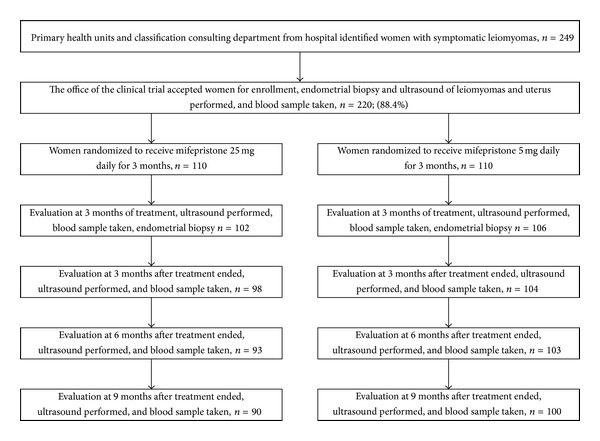
Flow chart for the trial.

All told, 249 subjects were referred to the consultative research centre, 29 of whom did not meet the inclusion criteria, and thus 220/249 (88.4%) subjects were included in the clinical trial, 110 in each treatment group, all of them going on to take mifepristone. In total, 37/220 (16.4%) were referred from the hospital infertility practice. The 3-month treatment was completed by 208/220 (94.5%) subjects, 102 and 106 from the 2.5 and 5 mg mifepristone groups, respectively. Nonattendance and consequent untraceability were the case with 5 and 4 subjects in the 2.5 and 5 mg groups, respectively. Prior to termination of the 3-month treatment, 3 subjects in the 2.5 mg group decided to undergo surgery due to “not feeling well.” (See Figure 1).

### 3.2. Initial Variables and Comparison between Treatment Groups

In Table 1 the general characteristics of all subjects included in the clinical trial can be seen. There were no significant differences between the treatment groups for any of them. Diagnosis of infertility associated with fibroids was present in 18/110 (16.4%) and 19/110 (17.3%) subjects in the 2.5 and 5 mg mifepristone groups, respectively, *P* = 0.428. One single myoma was present in 43/110 (39.1%) and 41/110 (3.7.3%) subjects in the 2.5 and 5 mg mifepristone groups, respectively, *P* = 0.278. In total, in 95/220 (43.2%) subjects, 6/220 (2.7%) and 119/220 (54.1%) had subserous, submucous, and intramural myomas, respectively; there were no significant differences between the mifepristone groups, *P* = 0.995. 

### 3.3. Efficacy

After the 3-month treatment, fibroid volume was reduced by 27.9% (CI 95% 20–35) and 45.5% (CI 95% 37–62), in the 2.5 and 5 mg groups, respectively, *P* = 0.003. At the end of treatment there was no reduction in fibroid volume, compared with initial values, in 29/99 (29.3%) and 10/105 (9.5%) subjects in the 2.5 and 5 mg groups, respectively, *P* < 0.001. The fibroid had disappeared or was not measurable in 2/102 (2.0%) and 3/106 (2.8%) subjects in the 2.5 and 5 mg groups, respectively, once administration of mifepristone was finished. 

As the treatment was over, uterine volume, compared with pretreatment values, decreased by 18.2% (IC 95% 5–42) and 22.1% (IC 95% 10–33), *P* = 0.264. There was no reduction in uterine volume in 35/99 (35.4%) and 25/105 (23.8%) in the 2.5 and 5 mg groups, respectively, *P* = 0.035. In Tables 2 and 3 the comparisons of the fibroid and uterine dimensional changes during all the study evaluative periods as well as the percentage changes in these volumes can be seen.  Table 4 shows changes in fibroid symptom prevalence during study evaluative periods. 

In the 2.5 and 5 mg groups at the beginning there were 41/110 (37.3%) and 45/110 (40.9%) subjects with hemoglobin values (Hb) ≤ 10 g/L, respectively, *P* = 0.290. After 49-day treatment, there were already 15/102 (14.7%) and 7/106 (6.6%) subjects with hemoglobin levels (Hb) ≤ 10 g/L *P* = 0.029. Upon completion of treatment, there were 15/102 (14.7%) and 7/106 (6.6%) subjects with Hb ≤ 10 g/L, in the 2.5 and 5 mg groups, respectively, *P* = 0.023. 

### 3.4. Side Effects of Mifepristone

On termination of treatment, 83/106 (78.3%) and 102/109 (93.6%) subjects were amenorrheic in the 2.5 and 5 mg groups, respectively, *P* < 0.001. Hot flushes were reported at some stage by 10/106 (9.4%) and 17/109 (15.6%) subjects in the 2.5 and 5 mg groups, respectively, *P* = 0.086. Nausea was reported at some point by 2/106 (1.9%) and 4/109 (3.7%) subjects in the 2.5 mg and 5 mg groups, respectively, *P* = 0.214. Vomiting was reported by 1/106 (3.8%) and 3/109 (2.7%) subjects in the 2.5 and 5 mg groups, respectively, *P* = 0.163. A feeling of fatigue was experienced by 2/106 (1.9%) and 4/109 (3.7%) subjects in the 2.5 mg and 5 mg groups, respectively, *P* = 0.214.

Before being treated, 2 subjects assigned to the 2.5 mg group and one in the 5 mg group had ASAT and ALAT values higher than 46 IU (normal reference score), and these scores were 48.8, 48.9, and 47.1 IU, respectively. Both transaminases (ASAT Y ALAT) were raised in 8/102 (7.8%) and 2/106 (1.9%) subjects in the 2.5 mg and 5 mg groups, respectively, *P* = 0.022. There were 5/102 (4.9%) subjects in the 2.5 group with at least one of the two transaminases raised and likewise 5/106 (4.7%) subjects in the 5 mg group. In total, raised transaminases, one or the other or both, were the case in 13/102 (12.7%) and 7/106 (6.6%) subjects in the 2.5 mg and 5 mg groups, respectively, *P* = 0.067. The three cases with raised pretreatment values registered normal values at the end of treatment. At no moment did maximum values ever exceed 99 and 79 U/L, for ASAT and ALAT, respectively.

Between the beginning and the end of treatment some bleeding, in the form of stains, had been reported by 22/93 (23.7%) and 25/103 (24.3%) subjects in the 2.5 mg and 5 mg mifepristone groups, *P* = 0.460; the average number of days with stains was 8.1 ± 5.3 and 5.7 ± 5.5 in the 2.5 mg and 5 mg groups, respectively, *P* = 0.150. Between the beginning and the end of treatment some irregular bleeding was reported by 7/93 (18.3%) and 15/103 (14.6%) subjects in the 2.5 mg and 5 mg groups, *P* = 0.241; the average number of days of such bleeding was 4.0 ± 2.6 and 5.3 ± 3.6 in the 2.5 mg and 5 mg groups, respectively, *P* = 0.235. In total, over the 3 months of treatment, there was irregular bleeding (blood and/or spotting) in 28/93 (30.1%) and 25/103 (24.3%) participants in the 2.5 mg and 5 mg mifepristone groups, respectively, *P* = 0.179: the average number of days of bleeding and stains was 8.7 ± 6.7 and 8.9 ± 8.7 in the 2.5 mg and 5 mg mifepristone groups, respectively, *P* = 0.937.

Table 5 shows changes in endometrial thickness at the end of treatment and in the 9-month followup. 

#### 3.4.1. Endometrial Biopsy

A pretreatment endometrial biopsy was performed on 38/220 (17.3%) subjects: secretory endometrium was diagnosed in 25/38 (65.8%), proliferative endometrium in 11/38 (28.9%) participants; 1 biopsy was not useful and another was diagnosed as irregular endometrial maturing. At the 45-day posttreatment consultancy, an endometrial biopsy was performed on 14 subjects in each mifepristone group as they have endometrial thickness superior to 8 mm, and 7 and 10 benign endometrial changes associated with the use of selective progesterone receptor modulators (SPRM) were diagnosed in the 2.5 and 5 mg mifepristone groups, respectively, *P* = 0.123. On termination of treatment, all patients were recommended to have an endometrial biopsy. In the 2.5 mg and 5 mg groups, respectively, 23/102 (22.5%) and 21/106 (19.8%) subjects did not undergo biopsy on their own decision, 13/102 (12.7%) and 14/106 (13.2%) biopsies were unsuitable for diagnosis. With regard to the remaining biopsies, there were 25/66 (37.9%) and 35/68 (51.5%) diagnoses of SPRM, *P* = 0.057, in the 2.5 mg and 5 mg groups, respectively. In total, the other diagnoses of secretory or proliferative endometrium were 24/134 (17.9%) and 50/134 (37.3%), respectively. There was no diagnosis of simple endometrial hyperplasia. 

### 3.5. Posttreatment Followup

Three months after the end of treatment, the followup consultation was attended by 98/110 (89.1%) and 104/110 (94.5%) subjects in the 2.5 and 5 mg mifepristone groups, respectively. There were 4 followup dropouts in the 2.5 mg group: 1 simple dropout and 3 who decided to undergo surgery: one due to very heavy periods, another due to a very large fibroid, and the third gave no reason for her decision. In the 5 mg group, one subject abandoned the study for personal problems and another underwent a hysterectomy due to heavy bleeding and required a blood transfusion. At this visit, hemoglobin levels below 10 mg/dL were registered by 20/98 (20.4%) and 7/104 (6.7%) subjects in the 2.5 and 5 mg mifepristone groups, respectively, *P* = 0.002. 

Six months after termination of treatment, the followup consultation was attended by 93/110 (84.5%) and 103/110 (93.6%) subjects in the 2.5 and 5 mg mifepristone groups. Absences were due to 1 subject in the 2.5 mg group who did not attend again and 1 who experienced a lot of pain and bleeding and wanted to undergo surgery. In the 5 mg group one subject dropped out claiming she felt “the same as before.” At this visit, hemoglobin was below 10 mg/dL in 16/93 (17.2%) and 6/103 (5.8%) subjects in the 2.5 and 5 mg mifepristone groups, respectively, *P* = 0.006. 

Nine months after treatment, the followup consultation was attended by 90/110 (81.8%) and 100/110 (90.1%) subjects in the 2.5 and 5 mg mifepristone groups, respectively. In the 2.5 mg group, 2 stopped attending visits and 1 wished to have surgery. In the 5 mg group, 3 subjects dropped out of the study: one did not attend, 1 underwent surgery due to heavy bleeding, and 1 presented an ovarian cyst. At this visit hemoglobin was below 10 mg/dL in 19/90 (21.1%) and 10/100 (10.0%) subjects in the 2.5 and 5 mg mifepristone groups, respectively, *P* = 0.017.

In total, throughout the study, including the followup period, dropout figures were 20/110 (18.2%) and 10/110 (9.1%) in the 2.5 and 5 mg mifepristone groups, respectively, *P* = 0.025.

In total, during the posttreatment observation period, there were 6 pregnancies, 2 in the 2.5 mg group and 4 in the 5 mg group. Two of the 4 pregnant subjects in the 5 mg group had been diagnosed with fibroid-associated infertility. One pregnant subject in the 2.5 mg group had a voluntary abortion. 

## 4. Discussion

This is the first study to administer mifepristone for only 3 months and to carry out a posttreatment followup three times the length of the treatment period, that is, 9 months, given that the other studies' followups were restricted to only double the treatment time [8, 10, 18–21]. 

It is striking that the proportion of subserous fibroids; 48/100 (43.6%) and 47/100 (43.2%), is somewhat higher compared to our previous studies and also quite a lot higher than the usual frequency for fibroids found here. We do not know why this is the case in this study. 

At the end of treatment, this study does in fact result in a significantly greater decrease in fibroid volume in the 5 mg group, *P* = 0.003, unlike the results of the previous study with 2.5 and 5 mg doses of mifepristone when the 2.5 mg dosage displayed a similar efficacy to that of 5 mg [22]. The 5 mg dose maintains its greater effectiveness during the first 3 months of followup, which is to say that the fibroid regrew less rapidly rather slowly in the 5 mg group, and this tendency continues up to 6 months after treatment as the fibroid volume reduction percentages were 17.6% and 27.7% in the 2.5 and 5 mg groups, respectively, with *P* values asymptotically significant. This difference disappeared 9 months after treatment. The greater effectiveness of the 5 mg dosage also shows up in the different percentages of cases where the fibroid volume remained unmodified: 29/99 (29.3%) and 10/105 (9.5%) in the 2.5 and 5 mg groups, respectively, *P* = 0.001. It should be pointed out that at the end of treatment the fibroid had disappeared or was unmeasurable in 2/102 (2.0%) and 3/106 (2.8%) subjects in the 2.5 and 5 mg groups, respectively. 

In this study, as was the case in the previous study with 2.5 and 5 mg doses of mifepristone [22], there was no significant decrease in uterine volume unlike in all the others when uterine volume reduction did occur although such decreases, ranging between 45 and 50%, were certainly less than those observed in fibroids. This uterine volume reduction takes place in all studies previously published by other authors [5–7, 12, 23, 24]. It is quite likely that this is due to the greater percentage of subserous fibroids in this study and although this type of fibroid also undergoes a decrease in volume since it is outside the uterus, measurements of the latter are not affected. Nevertheless, the uterine volume reduction percentage, using the 2.5 mg dosage, obtained at the end of treatment in this study (18.2%) is similar to the 11% reported by Eisinger et al. in their study [11].

Just as in our previous study with 2.5 and 5 mg mifepristone in which the subjects underwent surgery on completion of treatment (13), the percentage of women with anemia (Hb ≤ 10.0 g/L) was significantly less in the 5 mg group 45 days after treatment, and this continued to be the case until the end of the 9-month followup. This may well be connected to the significantly higher percentages of amenorrhea obtained in the 5 mg group. 

## 5. Symptoms

The two mifepristone groups are similar with respect to initial symptoms except for their urinary alterations being more frequent in those subjects receiving 2.5 mg. Although only rectal pain attained significant differences (*P* = 0.001), 49 days after treatment there was already a greater clinical effectiveness observable in the 5 mg group where most of the fibroid clinical data show asymptotically significant differences in favor of this dosage of mifepristone. This advantage disappears on termination of treatment but becomes significant in the sixth-month of followup when the signs and symptoms are significantly less prevalent in the 5 mg group and are even more accentuated 9 months after treatment (see Table 4). In other words, symptoms decrease faster and faster with the 5 mg dose and this improvement is more easily maintained for much longer than that with the 2.5 mg dosage. Regardless of the symptom prevalence 9 months after treatment in the 5 mg group, the absolute values of this prevalence in both groups continue to be significantly inferior to pretreatment values. 

## 6. Side Effects

There were no significant differences between the treatment groups with regard to hot flushes, nausea, vomiting, fatigue, and so forth, and the percentages obtained in this study remain within the levels registered in others [11, 22].

The percentages of subjects with raised transaminases are comparable with results reported in other studies [8, 10, 18–22]. In any case, no result exceeded 100 IU.

We did not observe significant differences between the two mifepristone groups vis-à-vis endometrial thickness, the averages of this variable being slightly superior to 8 mm, exactly as in our previous study with 2.5 mg of mifepristone [22]. 

There were no significant differences between the percentages of SPRM in the endometrial biopsies performed 45 days after treatment and in those done at the end of treatment; this difference was asymptotically significant, *P* = 0.057, which does not tally with the results obtained by Fiscella et al. who conclude that there are no differences in SPRM frequency when using doses of 2.5 and 5 mg of mifepristone and that perhaps lesser doses of 2.5 mg can be used. There was no diagnosis of simple hyperplasia in either of the 2 groups [25]. 

In both treatment groups, the initial UFS-QOL scores were similar. In the 2.5 mg mifepristone group, a significant improvement was apparent in all areas of the test except in that of “concern and activities.” In the 5 mg group, there was a significant improvement in all areas except “self-control.” On comparison of these results, there were no significant differences between the mifepristone groups neither in any area nor in the overall score (almost identical): 73.3 and 73.4 points, respectively, despite the relatively greater effectiveness of the 5 mg group. This result tallies with the one obtained by Fiscella et al. on the same subject [7]. 

It should be noted that 2 of the pregnancy cases had previous histories of fibroid-associated infertility.

The dropout figures of 20/110 (18.2%) and 10/110 (9.1%) in the 2.5 and 5 mg mifepristone groups, respectively, *P* = 0.025, were similar to those of our previous study of 2.5 versus 5 mg [22] and less than the 26% dropout figures of Eisinger et al. in his study of 2.5 mg mifepristone [11]. The higher number of dropouts in the 2.5 mg group might be due to the subjects experiencing a lesser, slower improvement in their symptoms. 

Internal validity: the random assignation resulted in homogenous treatment groups in every respect and avoided selection bias despite the study not being blind. The sample size was big enough to show up significant differences in fibroid volume reduction percentages. External validity: the clinical trial included 88.4% of the subjects sent to the recruitment consultancy, despite the fact that the inclusion and exclusion criteria could be considered very strict. Given that it was possible to treat almost 9 out of 10 subjects with symptomatic uterine fibroids, they represent the broad female population in a position to choose this treatment. Clinical relevance: the significant decrease in fibroid size, reduction in symptom prevalence, and intensity and the improvement in quality of life were clear enough to consider the results of the mifepristone treatment as being of clinical value.

By way of conclusion we could state that although the mifepristone doses studied were almost identical as indications of improvement of quality of life, it would be advisable to use the 5 mg dose given the significant improvement obtained in hemoglobin levels, the decrease or disappearance of hypermenorrhea and/or metrorrhagia, and the lower rate of dropouts. It is no coincidence that the dosage authorized by the European Medicine Agency for a drug chemically similar to mifepristone for presurgical treatment of uterine fibroids, named ulipristal acetate, is also 5 mg [26]. 

## Figures and Tables

**Table 1:** Characteristics of subjects by mifepristone treatment groups (Values are presented as average ± SD^+^ or *n* (%)).

Characteristics	2.5 mg	5 mg
	(*n* = 110)	(*n* = 110)
Age (years)	39.6 ± 6.0	39.0 ± 5.9
Body mass index (kg/m^2^)	24.9 ± 3.7	25.2 ± 4.0
Gravidity	2.7 ± 2.2	2.6 ± 2.2
Parity	0.6 ± 0.8	0.6 ± 0.7
Abortion	1.6 ± 1.9	1.4 ± 1.7
Fibroids volume (cc)	136 ± 129	112 ± 118
Uterine volume (cc)	455 ± 314	426 ± 305
Endometrial thickness (mm)	6.8 ± 1.9	6.8 ± 2.5
Hemoglobin (g/dL)	10.4 ± 1.7	10.5 ± 1.7
Aspartate aminotransferase (IU)	23.5 ± 9.0	22.9 ± 9.5
Alanine aminotransferase (IU)	20.7 ± 8.7	19.8 ± 9.2
Race		
White	32 (29.1)	27 (24.5)
Black	36 (32.7)	49 (44.5)
Afro-Cuban	42 (38.2)	34 (31.0)

^+^SD: Standard deviation.

**Table 2:** Changes in fibroid volumes (c.c.) by treatment groups and evaluation periods.

Evaluation at	Group	*n*	Mean ± S.D.	95% CI for mean	Changes (%)	P*
Before treatment	2.5 mg	110	136 ± 129	105–162	—	.151
	5 mg	110	112 ± 118	72–115		
3-month treatment	2.5 mg	102	98 ± 107	76–119	27.9 ↓	.003
	5 mg	106	60 ± 67	47–74	46.4 ↓	
3-month followup	2.5 mg	98	115 ± 144	85–153	15.4 ↓	.052
	5 mg	104	80 ± 109	51–84	28.6 ↓	
6-month followup	2.5 mg	93	112 ± 141	88–154	17.6 ↓	.086
	5 mg	103	81 ± 110	48–88	27.7 ↓	
9-month followup	2.5 mg	90	129 ± 157	97–167	5.1 ↓	.105
	5 mg	100	99 ± 91	69–118	11.6 ↓	

*t-test (ANOVA); CI: confidence interval; SD: standard deviation.

**Table 3:** Changes in uterine volumes (c.c.) by treatment groups and evaluation periods.

Evaluation at	Group	*n*	Mean ± S.D.	95% CI for mean	Changes (%)	P*
Before treatment	2.5 mg	110	455 ± 314	372–492	—	.486
	5 mg	110	426 ± 305	347–467		
3-month treatment	2.5 mg	102	372 ± 272	324–433	18.2 ↓	.264
	5 mg	106	332 ± 243	282–378	22.1 ↓	
3-month followup	2.5 mg	98	418 ± 266	368–490	8.1 ↓	.349
	5 mg	104	379 ± 320	300–395	11.0 ↓	
6-month followup	2.5 mg	93	437 ± 274	386–510	4.0 ↓	.279
	5 mg	103	478 ± 255	291–670	12.2 ↑	
9-month followup	2.5 mg	90	495 ± 321	429–569	8.8 ↑	.888
	5 mg	100	489 ± 265	309–421	14.8 ↑	

*t-test (ANOVA); CI: confidence interval; SD: standard deviation.

**Table 4:** Prevalence of fibroid symptoms before, after treatment, 3, 6, and 9 month after treatment according to groups (Data are presented as *n* (%*)).

Fibroid symptoms	2.5 mg	5 mg	P**
Pelvic pain			
Before treatment	81 (73.6)	87 (79.1)	.171
3-month treatment	14 (13.7)	9 (8.5)	.114
3-month followup	21 (21.4)	24 (23.1)	.389
6-month followup	31 (33.3)	24 (23.3)	.060
9-month followup	33 (36.7)	25 (25.0)	.041
Pelvic pressure			
Before treatment	74 (67.9)	73 (65.8)	.369
3-month treatment	3 (2.9)	3 (2.8)	.481
3-month followup	8 (8.2)	8 (7.7)	.451
6-month followup	18 (19.5)	8 (7.8)	.008
9-month followup	19 (21.1)	10 (10.0)	.018
Urinary symptoms			
Before treatment	45 (41.3%)	35 (31.5)	.066
3 month treatment	3 (2.9)	3 (2.8)	.481
3-month followup	7 (7.1)	8 (7.7)	.454
6-month followup	12 (12.9)	7 (6.8)	.074
9-month followup	12 (13.3)	6 (6.0)	.042
Lumbar pain			
Before treatment	59 (54.1)	56 (50.5)	.292
3-month treatment	3 (2.9)	1 (1.9)	.147
3-month followup	4 (4.1)	9 (8.7)	.093
6-month followup	15 (16.1)	10 (9.7)	.089
9-month followup	17 (18.9)	11 (11.0)	.062
Rectal pain			
Before treatment	29 (26.6)	37 (33.3)	.138
3-month treatment	4 (3.9)	2 (1.9)	.190
3-month followup	2 (2.0)	3 (2.9)	.350
6-month followup	6 (6.5)	6 (5.8)	.427
9-month followup	5 (5.6)	5 (5.0)	.432
Dyspareunia			
Before treatment	58 (53.2)	62 (55.9)	.347
3-month treatment	1 (1.0)	2 (1.9)	.300
3-month followup	5 (5.1)	8 (7.7)	.227
6-month followup	9 (9.7)	8 (7.8)	.318
9-month followup	12 (13.3)	6 (6.0)	.042
Hypermenorrhea			
Before treatment	79 (71.8)	73 (66.4)	.191
3-month treatment	4 (3.9)	2 (1.9)	.190
3-month followup	23 (23.5)	15 (15.4)	.073
6-month followup	38 (40.9)	21 (20.4)	<0.001
9-month followup	37 (41.1)	24 (24.0)	.005
Metrorrhagia			
Before treatment	15 (13.8)	22 (19.8)	.115
3-month treatment	0	0	—
3-month followup	2 (2.0)	0	.072
6-month followup	3 (3.2)	2 (1.9)	.285
9-month followup	2 (2.2)	1 (1.0)	.250

*Percentages on the number of subjects in each evaluative period (Figure 1).

**Normal approximation for proportions.

**Table 5:** Changes in endometrial thickness (mm) by treatment groups and evaluation periods.

Evaluation at	Group	*n*	Mean ± S.D.	95% CI for mean	Changes (%)	P*
Before treatment	2.5 mg	110	6.8 ± 1.9	6.5–7.3	—	1.000
	5 mg	110	6.8 ± 1.9	6.4–7.3		
3-month treatment	2.5 mg	102	8.9 ± 3.7	7.9–9.4	30.9 ↑	.846
	5 mg	106	9.0 ± 3.7	8.4–10.0	32.3 ↑	
3-month followup	2.5 mg	98	7.7 ± 2.3	7.1–8.2	13.2 ↑	.758
	5 mg	104	7.8 ± 2.3	7.3–8.3	14.7 ↑	
6-month followup	2.5 mg	93	7.7 ± 2.4	7.2–8.3	13.2 ↑	.594
	5 mg	103	7.9 ± 2.8	7.3–8.3	16.2 ↑	
9-month followup	2.5 mg	90	7.5 ± 1.9	7.1–7.9	10.3 ↑	1.000
	5 mg	100	7.5 ± 2.5	7.0–8.0	10.3 ↑	

*t-test (ANOVA); CI: confidence interval, SD: standard deviation.
